# *Tecticornia* sp. (Samphire)—A Promising Underutilized Australian Indigenous Edible Halophyte

**DOI:** 10.3389/fnut.2021.607799

**Published:** 2021-02-05

**Authors:** Sukirtha Srivarathan, Anh Dao Thi Phan, Hung Trieu Hong, Elvis T. Chua, Olivia Wright, Yasmina Sultanbawa, Michael E. Netzel

**Affiliations:** ^1^ARC Industrial Transformation Training Centre for Uniquely Australian Foods, Queensland Alliance for Agriculture and Food Innovation, The University of Queensland, Coopers Plains, QLD, Australia; ^2^Department of Biosystems Technology, Faculty of Technology, University of Jaffna, Kilinochchi, Sri Lanka; ^3^Algae Biotechnology Laboratory, School of Agriculture and Food Sciences, The University of Queensland, St. Lucia, QLD, Australia; ^4^School of Human Movement and Nutrition Sciences, The University of Queensland, St. Lucia, QLD, Australia

**Keywords:** halophyte, samphire, Australian indigenous, salinization, nutritional composition, phytochemicals, food

## Abstract

Salinization is gradually increasing over cropping soils and is challenging Governments in many countries, including Australia. There has been a high demand for utilizing arid and semi-arid land for sustainable food production. Currently, the main crops and forage plants are salt sensitive, while halophytes can tolerate a wide range of salinities. Samphire is an Australian indigenous edible halophyte and belongs to the genus *Tecticornia*. It is an underutilized, succulent plant growing on arid or semi-arid land. Most samphire species have a long history of use as food, but also as non-food (fodder and medicine), among indigenous communities in Australia, while scientific information is limited on their nutritional composition and potential bioactivity. The present study reports, for the first time, the nutritional composition, bioactive compounds (phytochemicals) and antioxidant capacity of six Australian grown samphire from different locations. The results showed that celosianin II and isocelosianin II could be identified as the predominant betalains (phytochemicals) in pigmented samphire species. Proximates and fiber varied significantly (*p* < 0.05) between the samphire species with a highest value of fiber of 46.8 g/100 g dry weight (DW). Furthermore, samphire could be identified as a valuable source of essential minerals and trace elements, such as iron (41.5 mg/100 g DW), magnesium (1.2 g/100 g DW) and sodium (16.7 g/100 g DW). The fatty acid profile, mainly palmitic, stearic, oleic, linoleic and α-linolenic acid, was similar among the studied species. Total phenolic content and DPPH-radical scavenging capacity were different (*p* < 0.05) between the six samphire samples. These initial results are very promising and indicate that Australian grown samphire may have the potential to be utilized as a functional food ingredient.

## Introduction

Throughout history, humans have attempted to utilize various natural produce for food solely to satisfy hunger (an essential need) at first; later they had choices to select in times of surplus, and they learnt how to produce food in terms of cooking and/or preservation through evolution (1, 2). Plants or plant-based products were gathered by First Peoples with various objectives such as food, folk medicine, clothes and rituals. Until agriculture was invented, humans were identified as hunter-gatherers during the paleolithic era. They mostly had a vast knowledge about native edible plants, varieties and variety of applications in the then modernized civilization. Unfortunately, this knowledge has gradually declined over time after a few generations of acculturation in Indigenous or Aboriginal Communities in various parts of the world (3). Today, most native edible plants are underutilized even though they have immense nutritional value for the community, while they may be still consumed in other parts of the world.

Underutilization of natural resources, the salinity of soil and water along with food insecurity, has become a major concern worldwide. It has been reported that 20% of the total farming land (45 million ha) are salt affected worldwide (4). It was estimated that salinity affects ~52.7 million ha (Mha) in Asia, 14.8 Mha in Africa, and 0.9 Mha in Australia (5). According to the National Land and Water Resources Audit (2001), ~1.8 Mha corresponding to 10% of the total farmed area is affected by dryland salinity in South-Western Australia, and another 6 Mha is at risk (6). Therefore, there is a high potential for utilizing saline lands for sustainable food production in the future.

Genus *Tecticornia*, Australian indigenous edible halophytes, commonly recognized as samphires, comprise 44 species endemic to Australia (7–9). *Tecticornia* sp. is an underutilized, succulent plant distributed in arid or semi-arid lands belonging to the subfamily Salicornioideae of the family Amaranthaceae. This family includes several striking economically important food crops such as spinach, beets, chard and quinoa (10). Remarkably, the subfamily Salicornioideae encompasses 110 species in 11 genera including *Sarcocornia* and *Salicornia* (8) with a broad range of climatic adaptations. Notably, *Tecticornia* sp. also expresses high salinity tolerance and thrives in flood, saline and drought conditions like other halophytes.

Halophytes consist of 1% of the world's flora that can tolerate salinity stressed environments through various mechanisms, while 99% of other plant species are supressed (11). For instance, *Tecticornia* sp. possess a variety of adaptations such as formation of compatible solutes (glycinebetaine), adventitious root, accumulation of salt in the tissues and selectivity in shoots for K^+^ over Na^+^ (12–14) to survive in environments with higher degrees of salinity. Unfortunately, studies which have investigated *Tecticornia* sp. have been largely limited to salinity tolerance (12, 15, 16) and their nutritional profile and potential bioactivity have not been explored to the same extent as other samphire species (*Salicornia* and *Sarcocornia* sp.).

The fleshy leaves and young green shoots of samphire are crunchy in texture with fresh sea-salt flavor. It is still consumed by indigenous people as part of a meal or complimentary meal without any processing, or quickly blanched and tossed with olive oil, vinegar or lemon, added to a meal as a salt substitute or served with seafood. Though *Tecticornia* species have been utilized by the Indigenous Australians for centuries as food, animal feed and as conventional medicine, this is no longer reported as physical evidence. However, a few studies have explored its medicinal properties. Bhanuvalli et al. (17) have recently examined the diuretic, analgesic, and anti-inflammatory properties of *Tecticornia* species (formerly *Halosarcia* sp.), collected from India. Later, *Halosarcia indica* was utilized in the production of low salt dried fish with value addition (18).

Remarkably, a few important chemical compounds are limited to halophytes with high potential to be used as functional ingredients (19, 20), which make them even more appealing to investigate further on nutritional properties. Besides the ecological importance of *Tecticornia* sp., investigations regarding its potential as functional food or functional (food) ingredient are very limited. Therefore, to bridge this gap, the present study provides the first comprehensive analysis of the nutritional composition, bioactive compounds (phytochemicals) and antioxidant capacity of six Australian grown samphire collected from different sublocations in the Kimberley Region of Western Australia.

## Materials and Methods

### Materials

**Figure 1:**
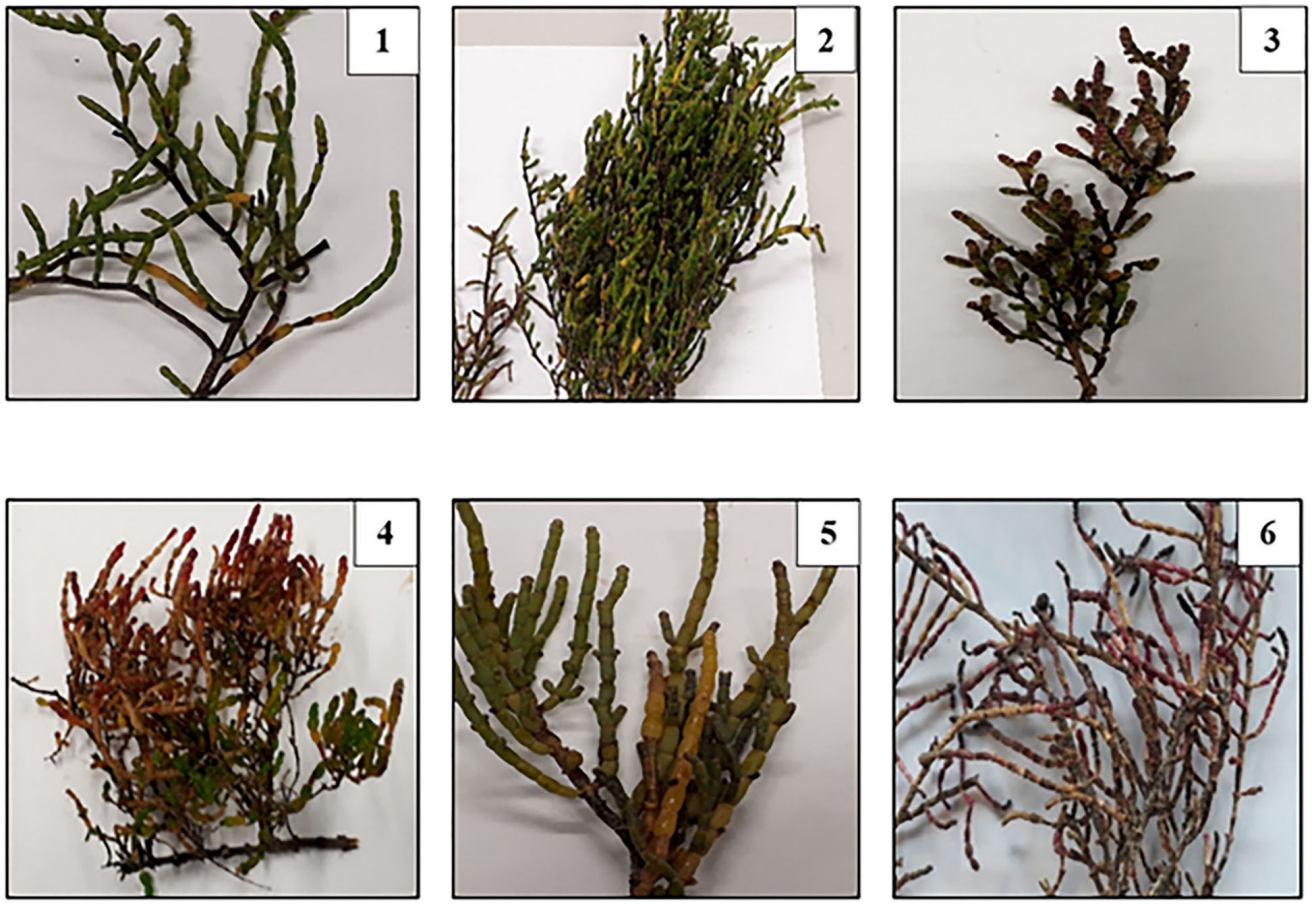
*Tecticornia* species collected from different sub-locations. 1: *T. halocnemoides*; 2: *T. halocnemoides*; 3: *T. halocnemoides*; 4: *T. halocnemoides*; 5: *T. indica*; 6: *T. halocnemoides*.

Fresh samples of *Tecticornia* sp. (Figure 1) were supplied by the Indigenous Community of Twin Lakes Cultural Park (Kimberley, WA) in 2019. The leaves and young twigs were freeze-dried and ground into a fine powder by using a MM 400 Retsch Mixer Mill (Retsch, Haan, Germany) and stored in airtight containers at −35°C for further analysis. All samples were identified and reference voucher numbers were given by the Queensland Herbarium, Botanic Gardens Mt Coot-tha, Brisbane, QLD, Australia (Table 1).

**Table 1:** Identified botanical specimens by Queensland Herbarium.

**Sample code**	**Identified name**	**Voucher reference no**.
UQ19DAM1	*Tecticornia halocnemoides*	AQ952399
UQ19DAM2	*Tecticornia halocnemoides*	AQ952394
UQ19DAM3	*Tecticornia halocnemoides*	AQ952395
UQ19DAM4	*Tecticornia halocnemoides*	AQ952396
UQ19DAM5	*Tecticornia indica*	AQ952401
UQ19DAM6	*Tecticornia halocnemoides*	AQ952424

### Reagents

Phenolic standards including gallic acid, 2,2-diphenyl-1-picrylhydrazyl (DPPH) and 6-hydroxy-2,5,7,8- tetramethylchromane-2-carboxylic acid (Trolox) (HPLC grade) were purchased from Sigma-Aldrich (Castle Hill, NSW, Australia). Betanin (>99% purity) was purchased from Adooq Bioscience LLC (Irvine, CA, USA).

### Proximate and Mineral/Trace Element Analysis

Proximate analysis was conducted (on the lyophilized powder) at the School of Agriculture and Food Sciences, The University of Queensland, St. Lucia, QLD, Australia. The analysis were conducted according to the Association of Official Analytical Chemists (21) methods as follows: dry matter (method 925.10), crude protein (method 990.03), crude fat by Soxhlet extraction (method 960.39), crude ash (method 923.03), and neutral detergent fiber (method 962.09). Soluble carbohydrates (glucose) and starch were measured using an enzymatic method (22, 23). Minerals, trace elements and heavy metals were determined by inductively coupled plasma optical emission spectrometry (ICP-OES) (24). The results of proximate composition and minerals were expressed as a percentage (%) on dry weight (DW) basis while trace elements and heavy metals were expressed as mg/kg DW.

### Fatty Acid Analysis

#### Lipid Extraction

The extraction of lipids followed Bligh and Dyer (25) and Ryckebosch et al. (26), with modifications. Briefly, ~0.2 g of dried sample material was mixed with methanol for 5 s using a vortex mixer (Fisher Biotech, Perth, WA, Australia). Then the samples were placed in a sonication bath (ELMA ultrasonic bath, Techspan, Brisbane, QLD, Australia) for 15 min at room temperature (RT). Chloroform (CHCl_3_) and Milli Q water were added to the sample and vortexed for 5 s. The sample was then centrifuged at 800 rpm for 5 min at RT (Eppendorf Centrifuge 5804, Eppendorf, Hamburg, Germany). The upper layer was removed, and the lower layer was collected into pre-weighed containers using a Pasteur pipette. The remaining pellet was re-extracted with chloroform and methanol mixture (1:1, v/v). The mixture was centrifuged at 800 rpm for 5 min at RT and the supernatant was collected and combined with the previously collected top layer. Finally, the combined solution was evaporated at 45°C under nitrogen flow. The extraction was performed in triplicate.

#### Fatty Acid Methyl Ester Analysis

The lipids and free fatty acids extracted from *Tecticornia* sp. were derivatized to the corresponding fatty acid methyl esters (FAME) and analyzed according to Chua et al. (27), with some modifications. In brief, 40 μL of the resuspended lipid solution (10 mg/mL in chloroform) was mixed together with 40 μL of heneicosanoic acid (C21) (2 mg/mL in isooctane) and 500 μL of 5% acetyl chloride in methanol in a thermal vial. Then the samples were heated at 95°C for 1 h and subsequently cooled to RT. After cooling, 500 μL of 0.9% NaCl solution and 500 μL of isooctane were added and vortexed for 5 s. Finally, 180 μL of the top (isooctane) layer was collected and transferred to a GC vial. Methyl nonadecanoate (C19) (20 μL, 1 mg/mL in isooctane) was added into the vials as an internal standard before injecting into a Shimadzu GCMS-TQ8040 system (Shimadzu Scientific Instruments, Sydney, NSW, Australia) using an Agilent DB-23 fused silica capillary column (60 m × 0.25 mm diameter *i.d*, 0.15 μm film thickness; Agilent Technologies, Santa Clara, CA, USA). Helium was used as a carrier gas at a constant linear velocity of 42.7 cm/s. The temperature of the injection port was set at 230°C, and 0.2 μL of the sample was injected in split mode with a split ratio of 10. The gradient temperature program was as follows: 70°C for 1 min, then increased to 170°C at 30°C/min, and an increase to 230°C at 3°C/min. The ion source and interface temperatures of the mass spectrometer were set at 200 and 230°C, respectively. The analysis was set to Q3 full-scan mode with a mass range of 89–400 m/z. The fatty acids were identified using a Supelco 37-component FAME mix standard (Sigma-Aldrich) and verified using the National Institute of Standards and Technology (NIST14) library.

### Determination of Total Phenolic Content and DPPH Radical Scavenging Capacity

#### Extraction

The extraction of the samples was performed as described by Hong et al. (28). Briefly, 0.5 g of dried powder of *Tecticornia* sp. was vortexed with 3 mL of 80% aqueous methanol containing 0.1 M HCl. Then the mixture was shaken using a reciprocating shaker (RP1812, Paton Scientific, Victor Harbor, SA, Australia) for 10 min at 200 rpm and centrifuged (Eppendorf Centrifuge 5804) at 3,900 rpm for 10 min at 4°C. The supernatant was collected and the residue was re-extracted with the extracting solvent, followed by ultra-sonication at 4°C, shaking and centrifugation as described above until the supernatant was colorless. Finally, the supernatants were combined and filtered through a 0.2 μm PP membrane filter prior the determination of the total phenolic content and DPPH radical scavenging capacity. All extractions were performed in triplicate.

#### Total Phenolic Content (TPC)

TPC was determined employing the Folin-Ciocalteu assay as described previously by Phan et al. (29), using a micro-plate absorbance reader (Sunrise, Tecan, Mannedorf, Switzerland) at 700 nm. TPC was expressed as milligrams of gallic acid equivalents per 100 grams of sample (mg GAE/100 g), based on an external gallic acid standard curve (0–105 mg/L).

#### DPPH Radical Scavenging Capacity

The methanolic sample extract was evaporated at 40°C, using a miVac sample Duo concentrator (Genevac Inc., Gardiner, NY, USA). The dried extract was re-dissolved in absolute methanol and further diluted to different concentrations for the DPPH assay.

The DPPH radical scavenging capacity was determined as previously described by Moore and Yu (30) with slight modifications using a microplate absorbance reader (Sunrise, Tecan) at 517 nm. The radical scavenging capacity was expressed as μM Trolox equivalents (TE) per g dry weight extract, based on an external Trolox standard curve (5–35 μM).

### Determination of Betalains

#### Extraction of Betalains

Powder samples (0.5 g) were vortexed with 3 mL of extractant (80% aqueous methanol containing 50 mM sodium ascorbate, pH 6.5) as reported previously by Schliemann et al. (31), with slight modifications. The sample mixture was shaken for 10 min at 200 rpm/min by using a RP 1812 reciprocating shaker (Paton Scientific, Victor Harbor, SA, Australia) followed by centrifugation (Eppendorf Centrifuge 5804) at 3,900 rpm for 10 min at 4°C. The supernatant was collected and the residue was re-extracted twice with 3 mL of the extractant. Supernatants were combined and filtered through a 0.2 μm PP membrane filter prior to betalain analysis. All extractions were performed in triplicate.

#### Analysis of Betalains

Compound separation and chromatographic analysis were performed on an Agilent 1290 Infinity ultra-high-performance liquid chromatography (UHPLC) system (Agilent Technologies, Waldbronn, Germany) equipped with a 1290 Infinity Diode Array Detector (DAD) and a reverse-phase Acquity UPLC BEH C18 column (150 × 2.1 mm i.d., 1.7 μm; Waters, Dublin, Ireland) maintained at 40°C. Mobile phases including A (96% MQ water, 3% acetonitrile, 1% formic acid v/v) and B (1% formic acid in acetonitrile v/v) eluted the compounds at a flow rate of 0.3 mL/min. The injection volume was 2 μL. The elution gradient was performed with 100% of mobile phase A for 1 min as an initial isocratic hold, then 96% A in 11 min and 84.8% A in 5 min, and from 84.8 to 10.9% in the next of 2 min. Then, an isocratic condition was used for 2 min, conditioning 1 min and re-equilibration for 5 min with 100% mobile phase A. DAD spectrum was scanned from 190 to 600 nm. The detection signal was recorded and quantified at 535 nm using betanin as an external standard.

A DIONEX Ultimate 3000 UHPLC system equipped with a UV/Vis detector and a Q Exactive high resolution Quadrupole-Orbitrap mass spectrometer (Thermo Fisher Scientific Australia Pty Ltd., Melbourne, VIC, Australia) was used to confirm the identity of the eluted betalains. The Q-Exactive mass spectrometer was operated in positive mode with full MS and all-ion-fragmentation (AIF) scans at a resolving power of 70,000 full width half maximum, at collation energy of 35 eV. A scan range of 100–1,000 m/z and 80–1,000 m/z was applied for the full MS and AIF scans, respectively. The automatic gain control (AGC) was set at 3e6. Chromatography and elution program were the same as those used for the UHPLC-DAD analysis described above. Peak identities were based on data regarding mass spectrum, fragmentations, calculated accurate mass, and retention time of betalain compounds and compared to previously reported literature. The Thermo Xcalibur^TM^ software (Thermo Fisher Scientific) was used for data acquisition.

### Analysis of Vitamin C

Ascorbic acid (L-AA) extraction and analysis was conducted as previously described by Phan et al. (32). Briefly, 0.5 g dried powder of *Tecticornia* sp. was extracted with 3% meta-phosphoric acid containing 8% acetic acid and 1 mM ethylenediamine-tetraacetic acid (EDTA). The reduction of dehydroascorbic acid (DHAA), which was also present in the extracts/samples, to L-AA was performed prior to UPLC-PDA analysis. Total vitamin C (L-AA + DHAA) was determined using a Waters UPLC-PDA system (Waters, Milford, MA, USA) and a Waters HSS-T3 column (Waters, Rydalmere, NSW, Australia) (100 × 2.1 mm i.d; 1.8 μm; 25°C), with aqueous 0.1% formic acid as the mobile phase (0.25 mL/min) and isocratic elution. An external calibration curve of L-AA was used for quantification.

### Determination of Anti-nutritional Components

#### Hydrolysable Tannins

The hydrolysable tannins in the *Tecticornia* sp. were determined using the potassium iodate assay previously described by Hoang et al. (33). Briefly, 50 μL extract was added to a 96-well plate with 150 μL of 2.5% w/v potassium iodate. Absorbance was measured after 15 min, using a Tecan microplate absorbance reader (Tecan Infinite M200, Mannedorf, Switzerland) at 550 nm. Tannic acid was used as a standard and results were expressed as mg tannic acid equivalents (TAE)/g DW.

#### Total Saponins

Extraction and quantification of saponins followed the spectrophotometric method described by Phan et al. (34) with modifications. Approximately 0.5 g dried powder of *Tecticornia* sp. was extracted with 10 mL of 80% methanol. Then the mixture was shaken using a reciprocating shaker (RP1812, Paton Scientific) for 1 hr at 200 rpm followed by ultra-sonication at RT and centrifuged (Eppendorf Centrifuge 5804, Eppendorf, Hamburg, Germany) at 3,900 rpm for 5 min at RT. The supernatant was collected and the residue was re-extracted with the extracting solvent while shaking on the reciprocating shaker at 150 rpm overnight. The supernatant was collected after centrifugation (3,900 rpm, 10 min), while the residue was re-extracted twice with 80% methanol (for 15 min). The supernatants were combined and evaporated until dryness at 40°C in a miVac sample Duo concentrator. The dried extract was redissolved in water and successively extracted with diethyl-ether to remove the pigments, followed by extraction of saponins with saturated n-butanol. The n-butanol extracts were combined and dried under reduced pressure using a rotary evaporator (Buchi Rotavapor R-100, BÜCHI Labortechnik AG, Flawil, Switzerland). The dried extract was redissolved in aqueous methanol 80% (v/v) and subjected to the Vanillin-H_2_SO_4_ assay using a microplate reader (Sunrise, Tecan) at 544 nm. Oleanolic acid (0–1.0 g/L) was used to prepare an external calibration curve. Total saponins were expressed as mg of oleanolic acid equivalents (OE) per 100 g of sample.

#### Phytate Content

Total phytate content was determined according to Joshi-Saha and Reddy (35). Approximately 0.5 g dried powder of *Tecticornia* sp. was added to 10 mL of 2.4% HCl in a centrifuge tube and shaken for 1 h. The mixture was then centrifuged at 10,000 × g at 10°C for 20 min. The supernatant obtained was mixed with 0.5 g NaCl in another centrifuge tube and vortexed for 60 s. The tube was kept in a freezer (−20 C) for 20 min, centrifuged at 10,000 × g at 10°C for 20 min and the clear supernatant obtained was used for the total phytate content assay. Briefly, 1 mL of the extract was mixed with 300 μL of Wade's reagent (0.03% FeCl_3_.6H_2_O + 0.3% sulfosalicylic acid). The mixture was centrifuged at 4,000 × g at 10°C for 5 min, and the absorbance was read using a microplate absorbance reader (Tecan Infinite M200) at 500 nm. Phytic acid (Inositol hexaphosphoric acid) dodecasodium salt (0–120 μg/mL) was used as a standard and results were expressed as mg/g of phytic acid (PA) on a dry matter basis (36).

#### Trypsin Inhibitor Activity Assay

The extraction and determination of trypsin inhibitor activity (TIA) was carried out using the method of Liu (37) with some modifications. Approximately 0.5 g dried powder of *Tecticornia* sp. was extracted with 25 mL NaOH. Then the mixture was shaken using a reciprocating shaker (RP1812, Paton Scientific) for 1 hr at 200 rpm. The mixture was allowed to settle for 10 min and the extract was carefully decanted without centrifugation for TIA estimation. For the TIA assay, an inhibitor assay buffer was prepared which contained 20 mM CaCl_2_ and 50 mM Tris-HCl at pH 8.2. The N-benzoyl-D-L- arginine- p-nitroanilide (DL-BAPA) substrate (0.4 mg/mL) was prepared fresh on the same day in the assay buffer that contained 1% dimethyl sulfoxide solution and pre-warmed at 37°C. An aliquot (2.5 mL) of the DL-BAPA substrate was added to 1 mL of the diluted extract after which 1.0 mL bovine trypsin (20 μg/mL in 1 mM HCl solution containing 5 mM CaCl_2_) was added and immediately mixed. The whole assay was conducted in a water bath at 37°C. Following incubation for 10 min at 37°C, the color reaction was terminated by addition of 0.5 mL of 30% acetic acid solution. The mixture was centrifuged at 3,000 × g for 10 min and the absorbance for the sample reading (A410S) at 410 nm was a measure of the trypsin activity in the presence of the sample inhibitors. The absorbance was read using a spectro-photo meter (Thermo Fisher Scientific Genesys 20, Melbourne, VIC, Australia) at 410 nm. Concurrently, the reaction was also run in the absence of inhibitors by replacing the sample extract with an equal amount of reverse osmosis (RO) water and reference reading was also recorded as A410R. Furthermore, sample blanks (A410SB) and reference blank (A410RB) were also run by adding the acetic acid solution before the trypsin solution. A trypsin unit is defined as an increase of 0.02 absorbance at 410 nm. The TIA is expressed in trypsin units inhibited (TUI) per mg sample and calculated as follows:

$$\begin{array}{l} \frac{TUI}{mg}=\frac{\left(CR-CS\right)\;x\;100\;x\;mL\;diluted\;extract}{mg\;sample\;per\;mL\;diluted\;extract\;used\;for\;the\;assay\;x\;2} \end{array}$$

Where CS (corrected sample reading) = A410S-A410SB.

CR (corrected reference reading) = A410R-A410RB.

### Statistical Analysis

The results were expressed as mean ± standard deviations (SD) and analyzed using a multi-variate general linear model (IBM SPSS statistics 26; IBM, Sydney, NSW, Australia). Pearson's correlation coefficient (R) and the coefficient of determination (R^2^) was calculated for testing the correlation between the DPPH radical scavenging capacity and TPC. The means were compared using ANOVA and Duncan's multiple range test, and probability was accepted at *p* < 0.05.

## Results and Discussion

### Proximate, Minerals, and Trace Elements

The results of the proximate composition, minerals and trace elements of the *Tecticornia* species collected from different sub-locations are presented in Table 2.

**Table 2:** Proximate composition, minerals and trace elements of *Tecticornia* species from different sub-locations.

**Plant species**	***T. halocnemoides* 1**	***T. halocnemoides* 2**	***T. halocnemoides* 3**	***T. halocnemoides* 4**	***T. indica* 5**	***T. halocnemoides* 6**	**Nutritional information**
**Proximate composition (g/100 g DW)**							
Protein	8.6 ± 0.0^b^	12.6 ± 0.1^d^	12.5 ± 0.1^d^	8.9 ± 0.1^c^	8.7 ± 0.1^b^	7.6 ± 0.1^a^	50 g^**^
Fat	1.76 ± 0.0^c^	1.77 ± 0.0^d^	1.86 ± 0.1^e^	1.84 ± 0.1^e^	1.07 ± 0.1^a^	1.45 ± 0.0^b^	70 g^**^
Glucose	5.1 ± 0.0^e^	5.6 ± 0.1^f^	2.7 ± 0.1^a^	4.6 ± 0.1^d^	4.0 ± 0.1^c^	3.5 ± 0.1^b^	90 g^**^
Starch	0.08 ± 0.1^c^	0.03 ± 0.0^ab^	0.09 ± 0.0^d^	0.2 ± 0.0^e^	0.04 ± 0.0^b^	0.03 ± 0.0^a^	310 g^**^
Fiber	35.1 ± 0.5^e^	30.8 ± 0.09^d^	30.1 ± 0.2^c^	27.8 ± 0.1^b^	46.8 ± 0.1^f^	26.4 ± 0.2^a^	30 g^**^
Moisture	59.0 ± 0.4^b^^*^	77.0 ± 0.5^de^^*^	71.7 ± 0.6^c^^*^	78.1 ± 0.5^e^^*^	75.5 ± 0.4^d^^*^	28.9 ± 2.1^a^^*^	-
Ash	1.0 ± 0.1^a^	1.2 ± 0.0^b^	2.6 ± 0.0^f^	2.0 ± 0.0^d^	2.4 ± 0.0^e^	1.5 ± 0.0^c^	-
**Minerals (g/100 g DW)**							
Ca	0.51 ± 0.0^e^	0.41 ± 0.0^c^	0.41 ± 0.0^c^	0.35 ± 0.0^a^	0.38 ± 0.0^b^	0.48 ± 0.0^d^	1.2 g/day AI^√^
Mg	0.8 ± 0.1^c^	0.9 ± 0.0^d^	0.59 ± 0.0^a^	1.2 ± 0.0^f^	0.63 ± 0.0^b^	1.1 ± 0.0^e^	0.35 g/day EAR^√^
Na	11.8 ± 0.0^c^	11.6 ± 0.0^b^	13.3 ± 0.1^d^	16.3 ± 0.1^e^	8.8 ± 0.0^a^	16.7 ± 0.1^f^	0.46–1.3 g/day AI^√√^
K	1.1 ± 0.1^b^	1.7 ± 0.1^e^	1.5 ± 0.0^d^	1.5 ± 0.0^d^	0.3 ± 0.0^a^	1.3 ± 0.0^c^	4.7 g/day AI^√^
P	0.12 ± 0.0^b^	0.13 ± 0.0^c^	0.2 ± 0.0^d^	0.12 ± 0.0^b^	0.12 ± 0.0^b^	0.07 ± 0.0^a^	0.7 g/day AI^√^
S	0.5 ± 0.1^b^	0.67 ± 0.0^d^	0.69 ± 0.0^e^	0.8 ± 0.0^f^	0.3 ± 0.0^a^	0.6 ± 0.0^c^	-
**Trace elements (mg/kg DW)**							
Fe	162.3 ± 3.0^c^	107.6 ± 4.2^ab^	112.9 ± 2.3^b^	160.9 ± 2.9^c^	101.4 ± 3.7^a^	414.6 ± 8.0^d^	8 mg/day RDA^√^
Zn	4.9 ± 0.1^b^	6.3 ± 0.2^c^	9.8 ± 0.3^d^	16.9 ± 0.3^e^	4.2 ± 0.4^a^	6.5 ± 0.3^c^	11 mg/day RDA^√^
Mn	11.3 ± 0.1^c^	15.3 ± 0.4^d^	10.8 ± 0.3^c^	44.1 ± 0.4^e^	4.9 ± 0.2^a^	9.7 ± 0.3^b^	2.3 mg/day AI^√^
Cu	2.9 ± 0.1^a^	7.0 ± 0.5^d^	10.5 ± 0.7^e^	6.9 ± 0.2^d^	3.7 ± 0.2^b^	5.4 ± 0.1^c^	900 μg/day AI^√^
Ni	0.03 ± 0.1^a^	1.5 ± 0.2^e^	0.6 ± 0.1^c^	0.9 ± 0.1^d^	0.8 ± 0.1^cd^	0.3 ± 0.0^b^	-
Mo	2.3 ± 0.1^d^	0.8 ± 0.1^a^	1.2 ± 0.1^c^	1.0 ± 0.1^b^	0.8 ± 0.1^a^	1.2 ± 0.1^c^	45 μg/day AI^√^
Se	0.1 ± 0.1^a^	0.1 ± 0.0^a^	0.33 ± 0.0^bc^	0.27 ± 0.0^b^	0.27 ± 0.1^b^	0.35 ± 0.1^c^	55 μg/day AI^√^
Sr	51.5 ± 0.1^d^	44.9 ± 1.4^c^	41.8 ± 1.5^b^	45.6 ± 0.9^c^	30.7 ± 1.5^a^	53.2 ± 0.9^d^	-
B	36.8 ± 0.1^a^	49.9 ± 1.6^d^	40.4 ± 0.8^b^	46.7 ± 2.1^cd^	79.0 ± 3.3^e^	44.2 ± 1.5^c^	-

*Values are means ± SD (n = 3); Means not sharing a common superscript (a–f) within a row are significantly (p < 0.05) different*.

* *-(g/100 g FW)*;

** *Daily Intake (38)*;

√ *Nutritional information (39); (-) not available; RDA, recommended dietary allowance; AI, adequate intake; EAR, estimated average requirement*;

√√ *Nutritional Information (40)*.

#### Moisture

The moisture content of the samples from sub-location 6 (28.9 g/100 g) was found to be lowest among the samples tested while the samples from sub-location 4 had the highest value (28.9 vs. 78.1 g/100 g, *p* < 0.05). Moisture content is crucial in terms of the physicochemical properties of processed foods, since low moisture content decreases the susceptibility for microbial growth and undesirable biochemical changes (41). The moisture content of 28.9% in the *Tecticornia halocnemoides* sp. is associated with minimum risk for microbial growth and shelf-life stability during storage.

#### Protein and Fat

The protein content of the studied *Tecticornia* species (samphire) ranged from 7.6 to 12.6 g/100 g DW. Since we report for the first time on the nutritional composition of *Tecticornia* sp., comparison with literature values for the same species could not be presented. However, when compared with other plants belonging to the subfamily Salicornioideae, the studied *Tecticornia* sp. had higher protein levels than *Salicornia ramosissima* (5.2 g/100 g DW), but lower levels than *Salicornia herbacea* (22.1 g/100 g DW) and comparable levels with *Salicornia bigelovii* (10.2 g/100 g DW) (42–44). A 100 g serve of *T. halocnemoides* sp. contributes to 15–25% of the daily intake of protein for adults [Food Standards Australia New Zealand (FSANZ)] (38). Regarding the fat content, *T. halocnemoides* collected from different sub-locations showed relatively similar values (1.5–1.9 g/100 g DW), and these were significantly (*p* < 0.05) different from *Tecticornia indica* (1.1 g/100 g DW). However, the fat content was similar to that previously reported for *S. ramosissima* (1.9 g/100 g DW) (42).

#### Ash and Fiber

The ash content of the samples collected from the 6 different sub-locations was different (1.0–2.6 g/100 g DW), reflecting the impact of the growing conditions. In contrast, the ash content of the genus *Tecticornia* was lower than that of *S. ramosissima* (29.2 g/100 g DW), *S. bigelovii* (52.7 g/100 g DW) and *S*. *herbacea* (8.1 g/100 g DW) (42–44). Interestingly, *Tecticornia* species are rich sources of fiber irrespective of their growing locations. The fiber content of the samples in the present study ranged from 26.4 to 46.8 g/100 g DW. *T. indica* (46.8 g/100 g DW) had a significantly (*p* < 0.05) higher fiber content than *T. halocnemoides* (26.4–35.1 g/100 g DW), collected from different sub-locations. The fiber content of the genus *Tecticornia* was surprisingly higher than that of the genus *Salicornia*. Although *S. ramosissima* (22.5 g/100 g DW) had slightly lower fiber, it can still be considered as a rich source of fiber according to Barreira et al. (42). As reported by FSANZ (38), the AI values of dietary fiber for males and females are 30 and 25 g per day, respectively. Thus, it is no surprise that consuming samphire with other meal components would be beneficial, since the high content of fiber would aid in improving gut health and digestion beyond the food's nutritional “standard” value. Overall, the studied samphire species have the potential to be used as low-energy foods since they are low in fat and carbohydrates, but high in dietary fiber and protein.

#### Minerals and Trace Elements

The most abundant minerals were sodium (Na), iron (Fe), magnesium (Mg), molybdenum (Mo) and manganese (Mn) in the genus *Tecticornia* (Table 2). The sodium content in 100 grams of the *Teticornia* species is substantially higher than the recommendations for daily sodium intake internationally (0.46–1.3 g per day) (40). Among the *Tecticornia* sp., *T. indica* (8.8 g/100 g DW) had the lowest amount of sodium, while *T. halocnemoides* ranged from 11.6 to 16.7 g/100 g DW, which could be related to the different growing conditions (sub-locations). Fe was also lowest in *T. indica* (101.4 mg/kg DW). In addition, the minerals in *Tecticornia* sp. were found at comparable levels with that reported for *S. ramosissima* (42). However, certain minerals were higher in *Tecticornia* sp., particularly calcium (Ca) and potassium (K). In general, the studied Australian indigenous edible halophytes are valuable sources of important minerals and trace elements. The high content of minerals present in halophytes is a result of the environment where they grow (high salinity) as well as their potential to “absorb” and accumulate these compounds as postulated by Díaz et al. (44).

#### Heavy Metals

Concerning the presence of heavy metals, the samples studied contained low concentrations of these elements (Table 3). This is important, as heavy metal intake can cause significant health complications (39, 49). Our findings are in agreement with the results reported by Barreira et al. (42). Nutritional information on heavy metals has been added to Table 3 for extrapolation. However, when plants like halophytes grow in polluted areas, they may accumulate heavy metals at higher concentrations, specifically in their roots.

**Table 3:** Heavy metals in *Tecticornia* species from different sub-locations.

**Plant species**	***T. halocnemoides* 1**	***T. halocnemoides* 2**	***T. halocnemoides* 3**	***T. halocnemoides* 4**	***T. indica* 5**	***T. halocnemoides* 6**	**Nutritional Information**
**Heavy metals (mg/kg DW)**							
Al	108.3 ± 0.0^d^	56.2 ± 1.5^bc^	57.0 ± 1.6^c^	51.3 ±0.8^a^	52.3 ± 0.3^ab^	130.5 ± 5.3^e^	1.0 mg/kg BW/week TWI^*^
As	0.1 ± 0.0^a^	0.2 ± 0.1^b^	0.2 ± 0.0^b^	0.1 ± 0.0^a^	0.3 ± 0.1^c^	0.2 ± 0.0^b^	128 μg/week for a 60 kg BW TWI^**^
Cd	0.15 ± 0.0^b^	0.15 ± 0.0^b^	0.23 ± 0.0^d^	0.19 ± 0.0^c^	0.1 ± 0.0^a^	0.1 ± 0.0^a^	2.5 μg/kg BW/week TWI^√^
Cr	0.93 ± 0.0^f^	0.5 ± 0.0^d^	0.05 ± 0.0^a^	0.12 ± 0.0^b^	0.4 ± 0.0^c^	0.87 ± 0.1^e^	35 μg/day AI^∧^
Pb	0.6 ± 0.0^b^	0.4 ± 0.1^a^	0.8 ± 0.1^c^	1.0 ± 0.1^c^	0.9 ± 0.1^c^	0.9 ± 0.2^c^	25 μg/kg BW/week TWI^¥^

*Values are means ± SD (n = 3); Means not sharing a common superscript (a–f) within a row are significantly (p < 0.05) different*.

* *European Food Safety Authority (EFSA) (45)*;

** *Leblanc et al. (46)*;

√ *EFSA (47)*;

∧ *Otten et al. (39)*;

¥ *Cheung Chung et al. (48); BW, body weight; TWI, tolerable weekly intake; AI, adequate intake*.

### Fatty Acids and Methyl Esters

**Figure 2:**
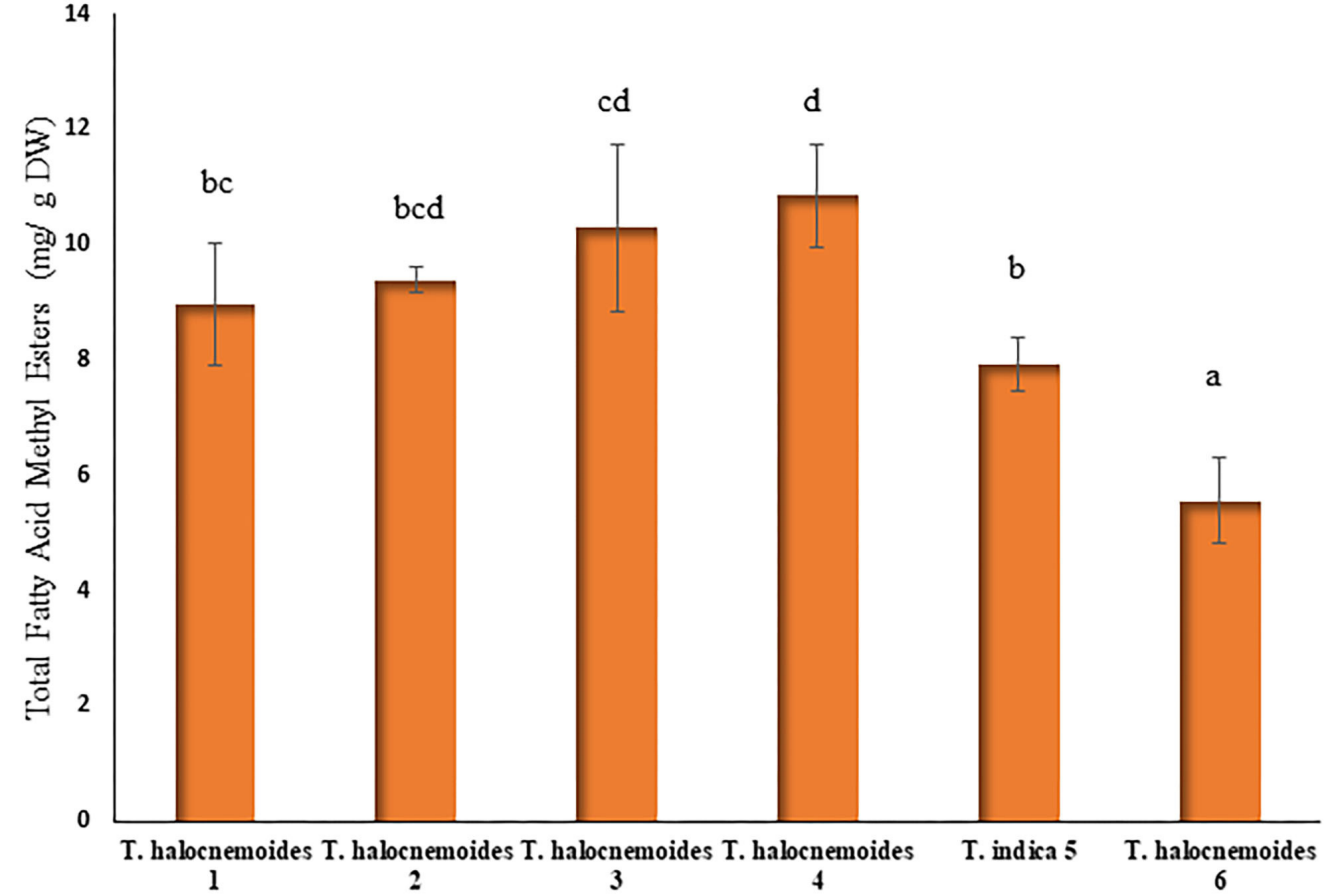
Total fatty acid methyl esters (FAME) concentration (mg/g DW) of *Tecticornia* species from different sub-locations. Data present means ± SD (*n* = 3). Different letters indicate significant differences (*p* < 0.05) in total fatty acid methyl esters among the samples tested.

The total lipid content (Figure 2) ranged from 5.6 to 10.8 mg/g DW which is comparable with the results (1.0–7.27 mg/g DW) reported for other halophytes (50). *Tecticornia halocnemoides* (10.8 mg/g DW; sub-location 4) had the highest lipid content, whilst *T. halocnemoides* (5.6 mg/g DW; sub-location 6) the lowest.

The saturated fatty acids (SFA) in *Tecticornia* species from different sub-locations ranged from 32.5% (*T. indica*) to 46.5% [*T. halocnemoides* (sub-location 6)] of the total fatty acids (Table 4). It is interesting that almost all the species had a SFA content < 40% of the total lipid content, except *T. halocnemoides* from sub-location 6. The most abundant SFA was palmitic acid (C16:0), which was found to be one of the prevalent fatty acids in other halophytes (50, 51). Besides palmitic acid, stearic acid was also found in those plant species studied. Furthermore, the fraction of monounsaturated fatty acids (MUFA) was significantly (*p* < 0.05) lower than that of SFA and polyunsaturated fatty acids (PUFA) in the studied *Tecticornia* species.

**Table 4:** Fatty acid profile, expressed as % of total fatty acids on dry weight basis, of *Tecticornia* species from different sub-locations.

**FA (%)**	**Common Name**	***T. halocnemoides* 1**	***T. halocnemoides* 2**	***T. halocnemoides* 3**	***T. halocnemoides* 4**	***T. indica* 5**	***T. halocnemoides* 6**
C16:0	Palmitic acid	29.5 ± 0.5^d^	28.2 ± 0.5^c^	27.4 ± 0.2^b^	29.5 ± 0.3^d^	24.7 ± 0.1^a^	35.5 ± 0.6^e^
C18:0	Stearic acid	7.6 ± 0.9^b^	5.3 ± 0.7^a^	5.9 ± 1.3^a^	6.8 ± 0.5^ab^	7.8 ± 0.2^b^	11.0 ± 1.3^c^
∑ SFA		37.1	33.5	33.3	36.3	32.5	46.5
C18:1 (*n*-9)	Oleic acid	20.3 ± 1.9^b^	15.0 ± 0.9^a^	16.8 ± 1.9^a^	20.1 ± 0.5^b^	19.6 ± 0.3^b^	23.5 ± 1.8^c^
∑ MUFA		20.3	15.0	16.8	20.1	19.6	23.5
C18:2 (*n*-6)	Linoleic acid	15.5 ± 1.5^a^	22.2 ± 1.7^b^	29.3 ± 2.8^c^	22.2 ± 1.0^b^	25.7 ± 0.6^b^	18.6 ± 2.7^a^
C18:3 (*n*-3)	α-linolenic acid	27.0 ± 0.8^d^	29.2 ± 0.4^e^	20.7 ± 0.3^b^	21.4 ± 0.4^bc^	22.2 ± 1.2^c^	11.4 ± 0.2^a^
∑ PUFA		42.5	51.4	50.0	43.6	47.9	30.0
∑ PUFA/∑ SFA		1.1	1.5	1.5	1.2	1.5	0.6
*n*-6/*n*-3		0.6	0.8	1.4	1.0	1.1	1.6

*Values are means ± SD (n = 3); Means not sharing a common superscript (a–e) within a row are significantly (p < 0.05) different*.

MUFA contributed 15% [*T. halocnemoides* (sub-location 2)] to 23.5% [*T. halocnemoides* (sub-location 6)] to the total fatty acids and oleic acid was found to be the only MUFA in the studied species (Table 4). Similar results were reported for *Carpobrotus edulis, Arthrocnemum macrostachyum*, and *S. maritima* (50, 52, 53). A significant (*p* < 0.05) variation in PUFA between the *Tecticornia* sp. could also be observed, with *T. halocnemoides* from sub-location 2 having the highest (51.4%) and *T. halocnemoides* (sub-location 6) the lowest (30%) content (Table 4).

Except *T. halocnemoides* (sub-location 6), all other samples contained linoleic acid, α-linolenic acid, and palmitic acid as major fatty acids. This was in agreement with previous studies on other halophytes such as C*rithmum maritimum* (51). The highest proportion (29.3%) of linoleic acid was found in *T. halocnemoides* (sub-location 3) and the lowest (15.5%) in *T. halocnemoides* (sub-location 1), whereas *T. halocnemoides* (sub-location 2) had the highest content of α-linolenic acid (29.2%).

Since vertebrates are not able to synthesize linoleic acid and α-linolenic acid, these PUFAs must be delivered through the diet (54). Furthermore, it is recognized that PUFAs provide health benefits, including anti-inflammatory activity, protection of the nervous system, delaying the onset of chronic diseases and are important for the proper functioning of proteins, enzymes and certain receptors (55–57). Particularly, linoleic acid was found to possess antimicrobial activity against fungi and potential preventative effects against specific cancers and atherosclerosis (58). All studied *Tecticornia* species had ratios of PUFA/SFA > 1 [except *T. halocnemoides* (sub-location 6)] with *T. halocnemoides* (sub-locations 2 and 3) and *T. indica* having the highest PUFA/SFA ratio of 1.5 (Table 4). It is suggested that the ratio of n-6/n-3 PUFA in foods should be < 4 (59) for optimum health. The n-6/n-3 PUFA ratios found in this study are within the values suggested.

### Total Phenolic Content

**Figure 3:**
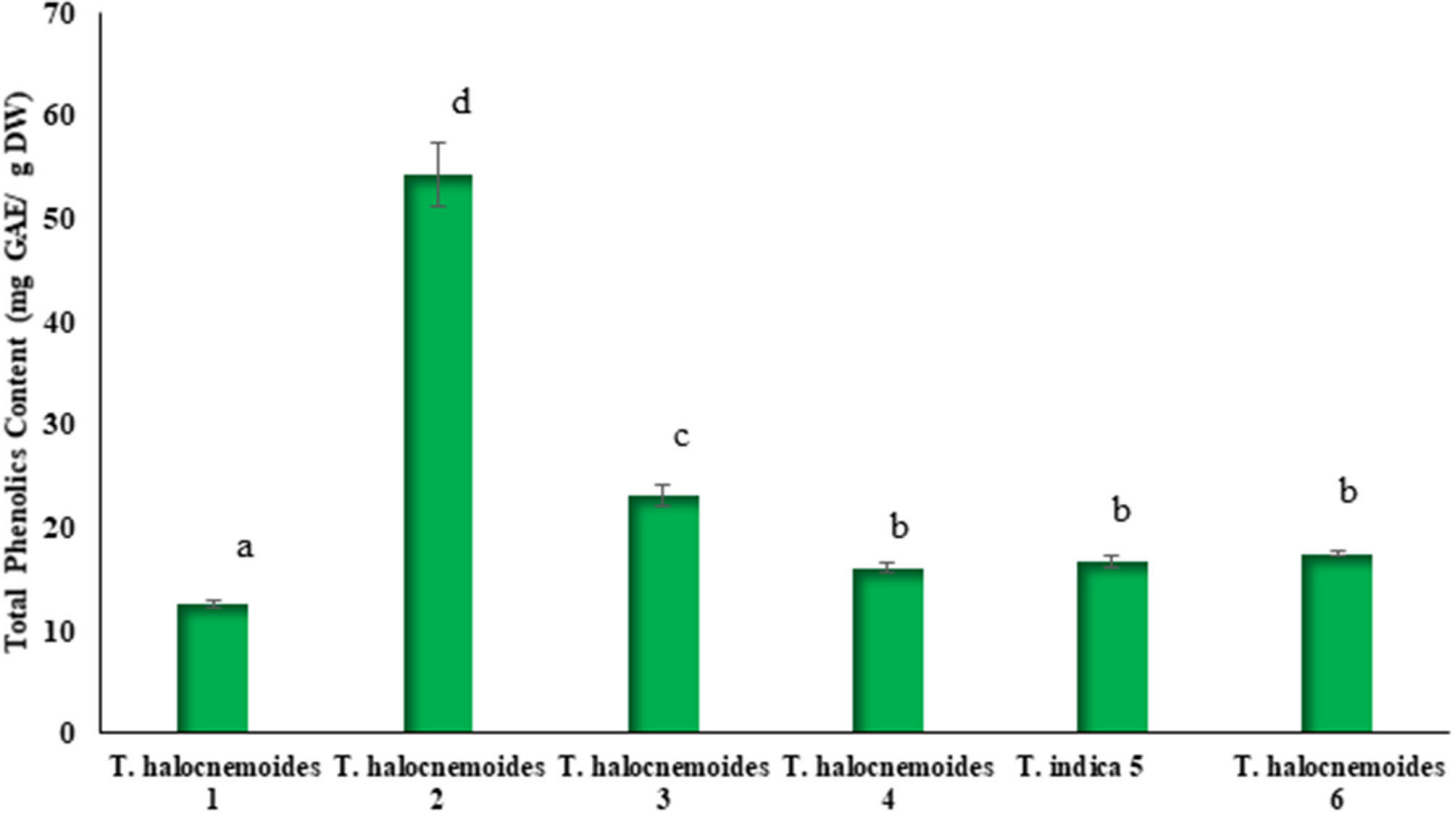
TPC of *Tecticornia* species from different sub-locations. Data present means ± SD (*n* = 3). Different letters indicate significant differences (*p* < 0.05) in TPC between the samples tested.

Figure 3 shows significant (*p* < 0.05) differences in TPC of the studied *Tecticornia* species. The TPC ranged from 12.6 to 54.2 mg GAE/g DW, with *T. halocnemoides* (sub-location 2) and *T. halocnemoides* (sub-location 1) having the highest and lowest values, respectively. *Tecticornia halocnemoides* (sub-locations 4 and 6) and *T. indica* had similar values while large variations in TPC content were observed among the same species of *T. halocnemoides* collected from different sub-locations (1, 2, and 3). The TPC in the *Tecticornia* sp. investigated in the present study was comparable with that of *S. ramosissima* and *S. fruticosa* (42, 60).

### DPPH Radical Scavenging Capacity

**Figure 4:**
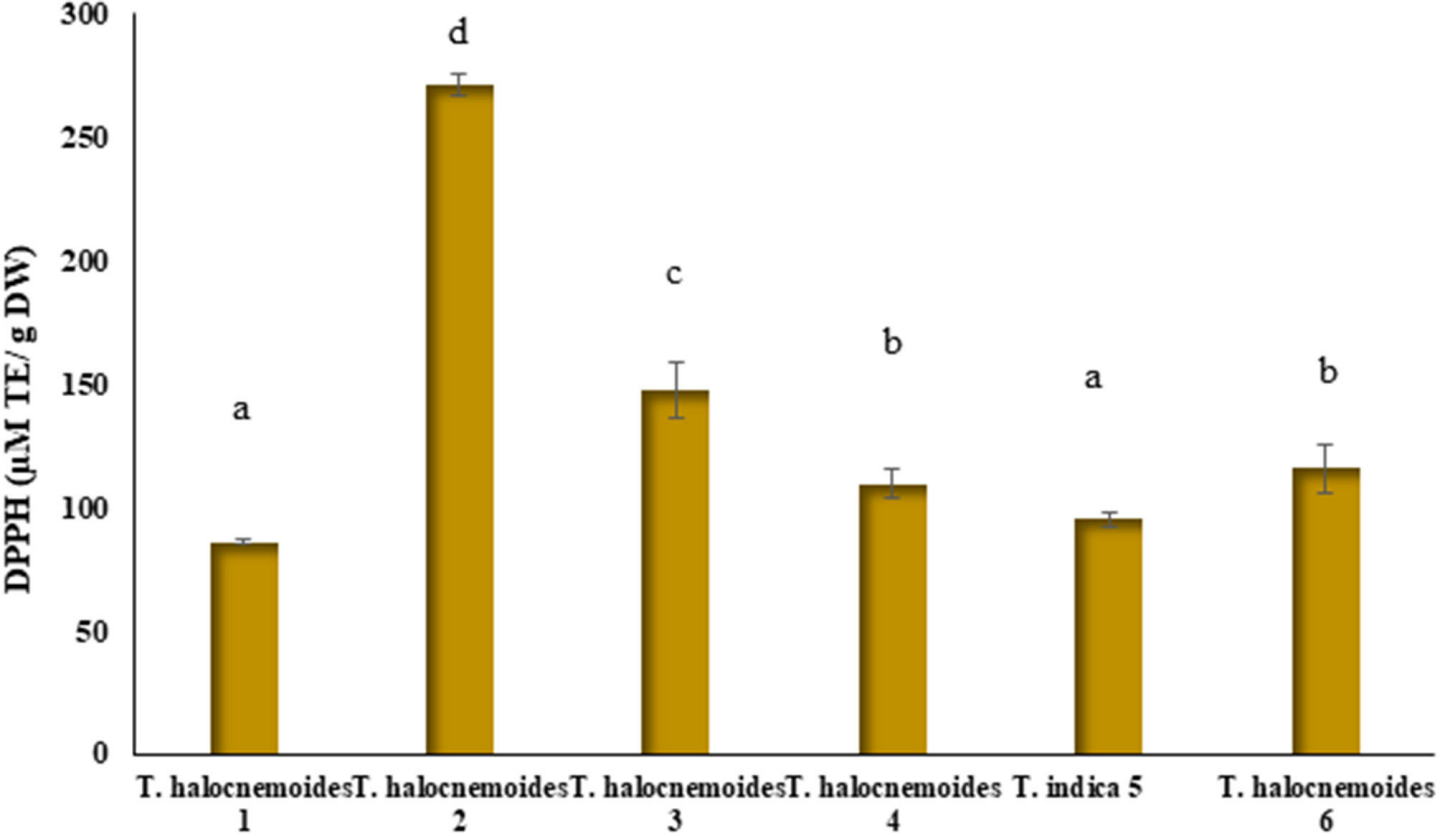
DPPH radical scavenging capacity of *Tecticornia* species from different sub-locations. Data present means ± SD (*n* = 3). Different letters indicate significant differences (*p* < 0.05) in DPPH radical scavenging capacity among the samples tested.

**Figure 5:**
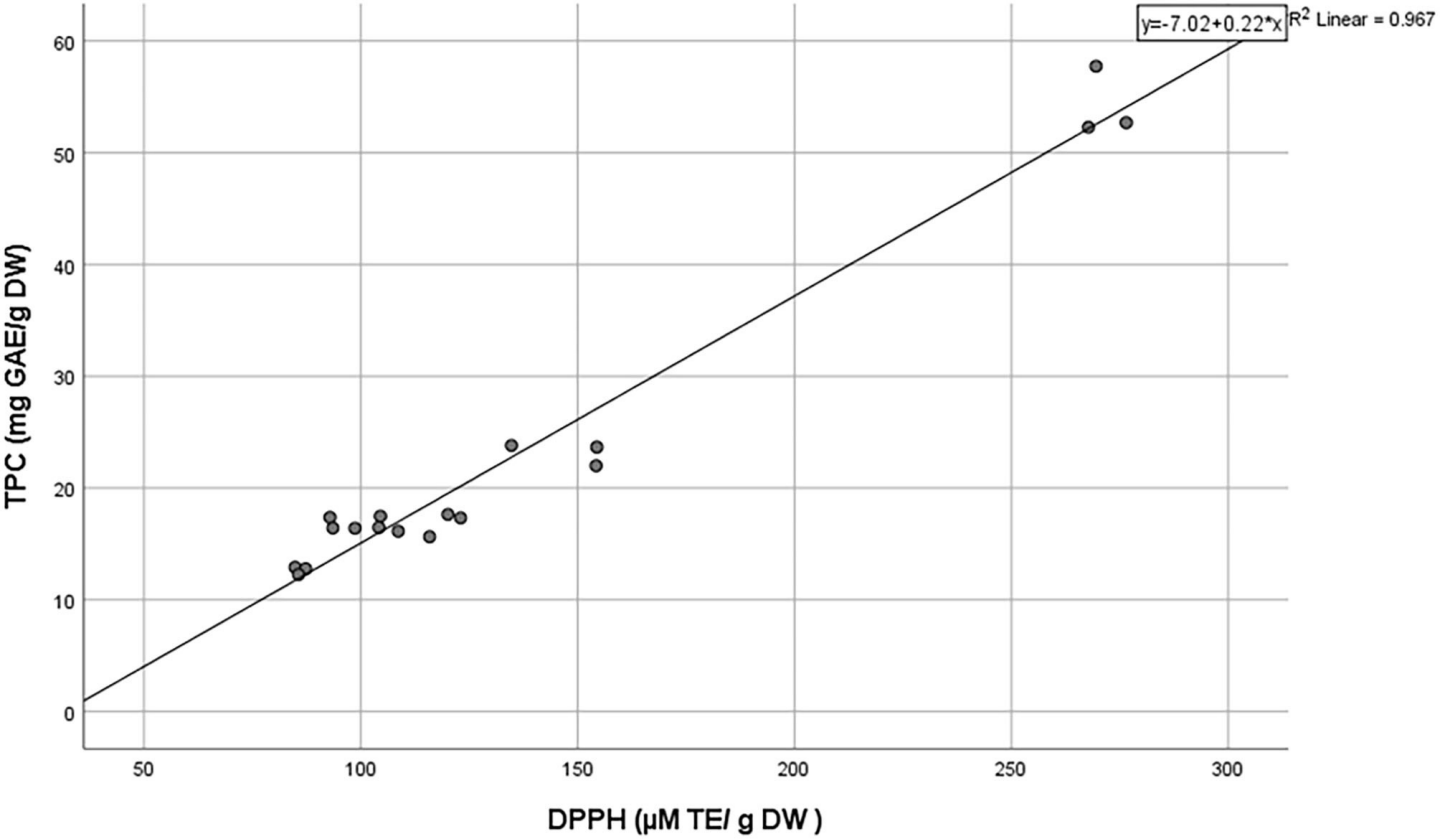
Correlation between TPC and DPPH radical scavenging capacity of *Tecticornia* species collected from different sub-locations.

Figure 4 shows significant (*p* < 0.05) differences in the DPPH radical scavenging capacity of the studied *Tecticornia* species. The highest radical scavenging capacity was determined in *T. halocnemoides* (sub-location 2) (271.3 μM TE/g DW) and the lowest in *T. halocnemoides* (sub-location 1) (85.9 μM TE/g DW), respectively. There was also a strong positive correlation between TPC and the DPPH values (R^2^ = 0.967; Figure 5). This finding is a strong indication that phenolic compounds are most likely the main antioxidants in the studied *Tecticornia* sp. However, the present results need to be interpreted with caution at this stage since only TPC data (spectro-photometrical assay) were determined.

### Betalains

#### Identification of Betalains

**Figure 6:**
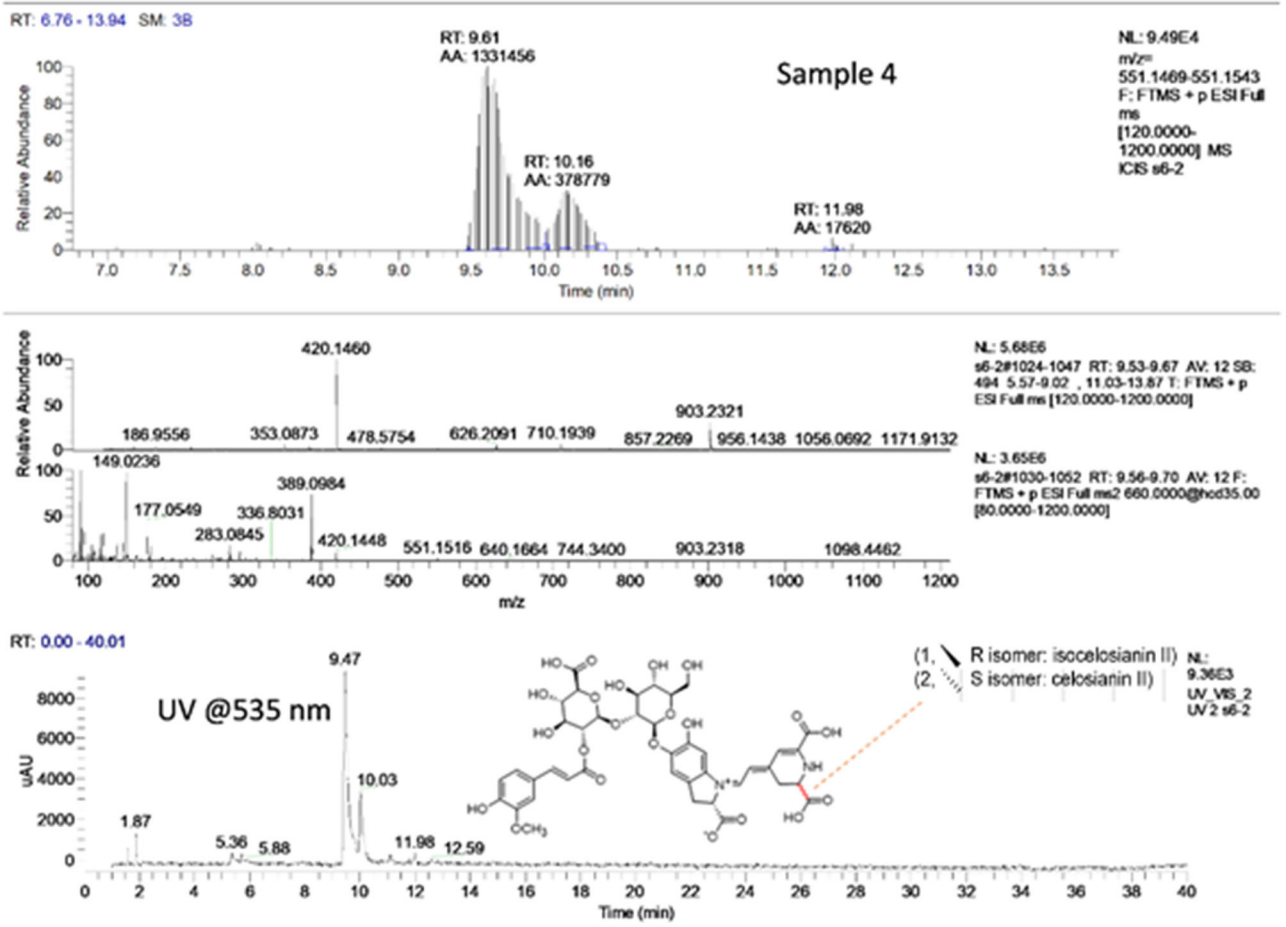
UHPLC-UV-MS/MS chromatograms of pigmented *T. halocnemoides* (sub-location 4) at 535 nm. Peak 1: celosianin II at 9.47 min and peak 2: isocelosianin II at 10.03 min.

The identification of betalains was based on their UV spectrum and their molecular masses determined by ultra-high-performance liquid chromatography with diode array detection and electrospray ionization-mass spectrometry (UHPLC-DAD-ESI-MS). Two main betalains, celosianin II and isocelosianin II (Figure 6) were identified in the pigmented *Tecticornia* sp.

An identical 2-peak profile was found in *T. halocnemoides* collected from sub-locations 2, 4, and 6. Peaks were identified by spiking experiments with betanin (commercial standard) and comparison with previously reported data (61, 62) (Table 5). As shown in Table 5, the identity (tentatively) of the two betalains in *T. halocnemoides* species were confirmed by using Q-Exactive high-performance quadrupole-Orbitrap high-resolution mass spectrometry. The MS spectra of peak 1 and 2 m/z 903.2321 [M+H]^+^ (calculated for C_40_H_42_O_22_N_2_, 903.2321) and its MS^2^ fragment patterns showed that the respective ion at m/z 903.2321 was fragmented to two product ions at 389.0984 and 551.1515, respectively, corresponding to betanidin (calculated for C_18_H_16_O_8_N_2_) and betanin (calculated for C_24_H_26_O_13_N_2_). Therefore, peak 1 and 2 are the signals of celosianin II and isocelosianin II, respectively. Interestingly, celosianin II was found in *Celosia cristata* (63), the plant having a similar color as the *T. halocnemoides* species. This study reported for the first time the presence of celosianin II and isocelosianin II as the main pigments in *T. halocnemoides*.

**Table 5:** Identification of betalain compounds in *Tecticornia* sp. using UHPLC-DAD-ESI-MS.

**Peak number**	**Retention time (min)**	**UV Max**	**HR-M^**+**^ ion**	**Formula**	**Identification**	**Plant**
1	9.61	535	903.23	C_40_H_42_O_22_N_2_	betanidin 5-O-(2″-O-E-feruloyl)-β-glucuronosyl-glucoside	*T. halocnemoides*
2	10.16	535	903.23	C_40_H_42_O_22_N_2_	isobetanidin 5-O-(2″-O-E-feruloyl)-β-glucuronosyl-glucoside	*T. halocnemoides*

Marchesini et al. (64) reported the pigment profile of *T. indica* and *T. auriculata* and also identified betalains as the main pigments/phytochemicals which is in agreement with the present study. It is interesting to note that the pigment concentrations in *Tecticornia* were more sensitive to seasonal changes (64). The large variation in the pigment profile of *Tecticornia* sp. probably reflects the differences in cultivars, locations and growing conditions. Therefore, further studies investigating the impact of these “parameters/variables” on the betalain profile and composition in Australian grown halophytes are necessary.

#### Betalain Content

**Figure 7:**
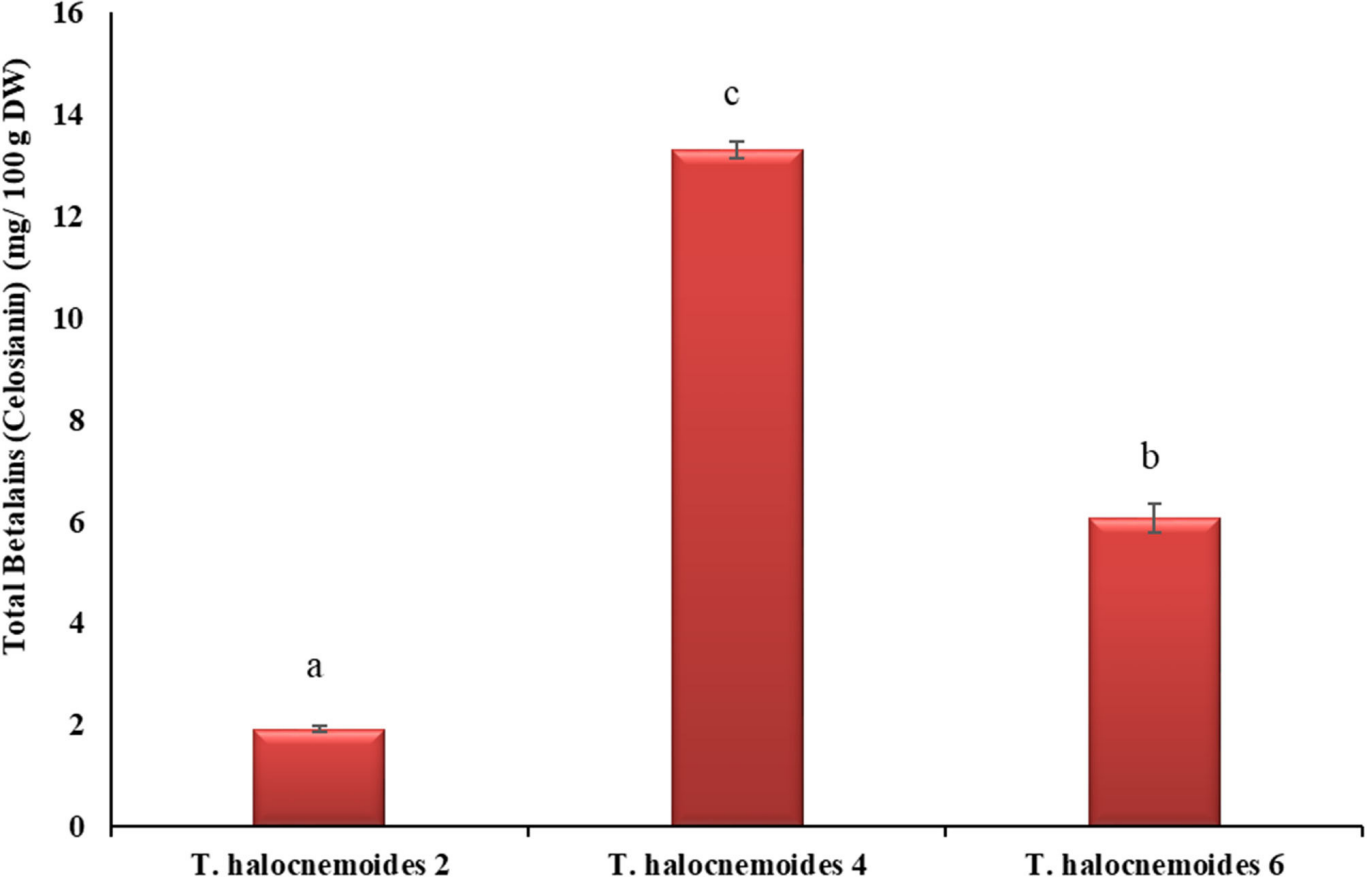
Betalains in pigmented *Tecticornia* species from different sub-locations (calculated as betanin equivalents (BE). Data present means ± SD (*n* = 3). Different letters indicate significant differences (*p* < 0.05) in total betalains among the samples tested.

The betalain content of the studied pigmented *Tecticornia* sp. ranged from 1.9 to 13.3 mg BE/100 g DW (Figure 7). Among the three *Tecticornia* sp. investigated, *T. halocnemoides* (4) had the highest (*p* < 0.05) betalain content (13.3 mg BE/100g DW) which is most likely caused by its reddish colored leaves and stems. These findings are comparable with previously reported concentrations for betalains in *T. auriculata* (20 mg/100 g DW), but lower than in *T. indica* (30 mg/100 g DW) (64). The observed differences to literature data may be caused by differences in harvest season and maturity stage as well as soil composition and growing conditions. It should also be noted that the betalain content can increase during stress induced by salinity or seasonal shifts (64).

**Figure 8:**
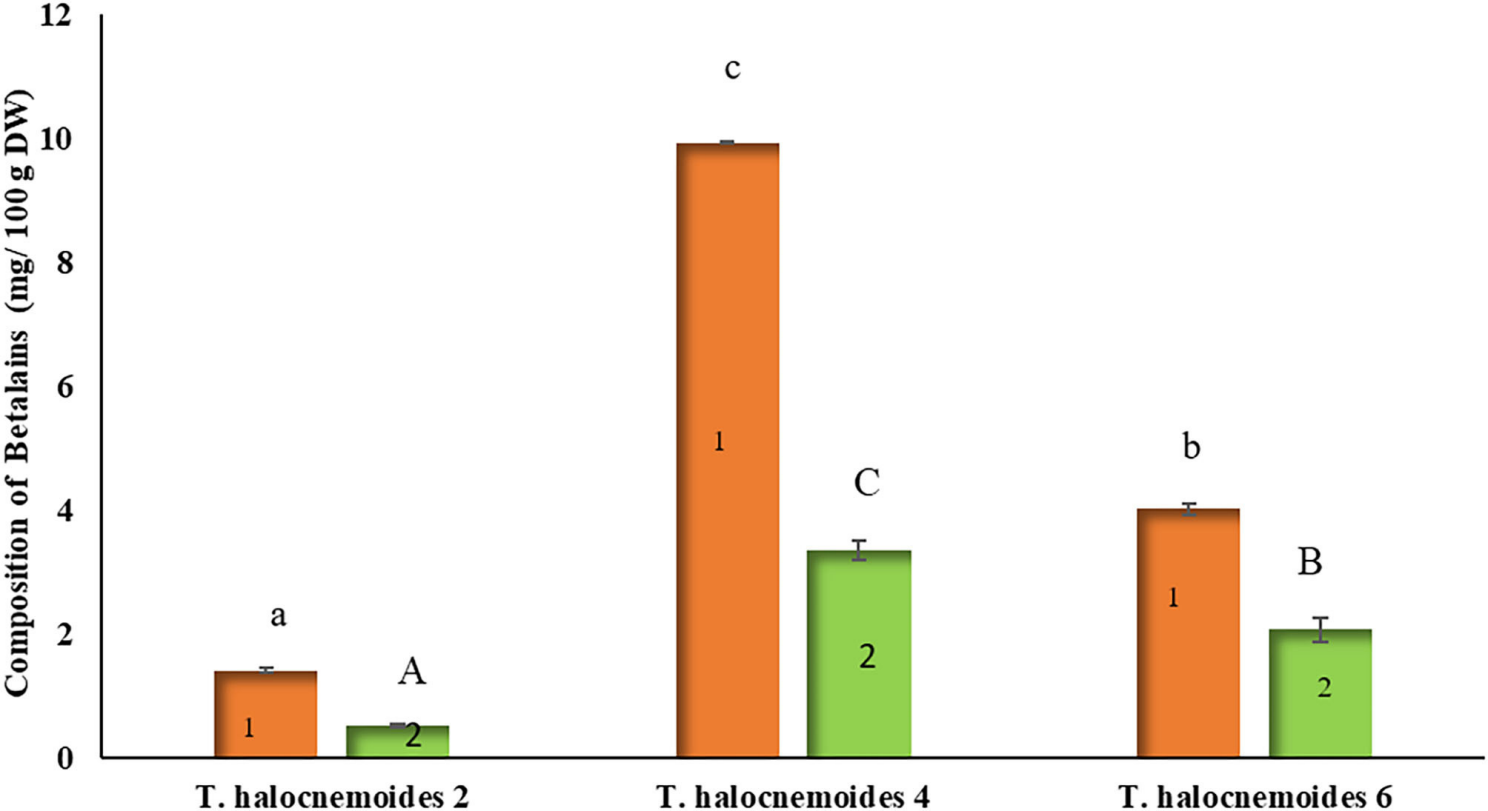
Individual betalains in pigmented *Tecticornia* sp. from different sub-locations (calculated as BE): (1) celosianin II; (2) isocelosianin II. Data present means ± SD (*n* = 3). Different letters indicate significant differences (*p* < 0.05) in composition of betalains among the samples tested.

Among the three pigmented *Tecticornia* species, celosianin II was the predominant betalain, ranging from 66.1 to 74.8% of the total peak area (Figure 8). The resulting concentrations of celosianin II, expressed as betanin equivalents (BE), in *T. halocnemoides* (sub-location 2), *T. halocnemoides* (sub-location 4) and *T. halocnemoides* (sub-location 6) were 1.4, 9.9 and 4.0 mg BE/100 g DW, respectively. Isocelosianin II was found in lower concentrations: 0.5 mg BE/100 g DW in *T. halocnemoides* (sub-location 2), 3.4 mg BE/100 g DW in *T. halocnemoides* (sub-location 4) and 2.1 mg BE/100 g DW in *T. halocnemoides* (sub-location 6).

Overall, the findings obtained in this study are consistent with what was reported by Marchesini et al. (64), regarding betalains in *Tecticornia* sp. It should be noted that betalains received significant attention due to their use as natural food colorants and their antioxidant and radical scavenging properties (65–67). Other biological activities such as inhibition of lipid peroxidation and LDL oxidation, prevention of DNA-damage, induction of antioxidant and phase II detoxifying enzymes, gene regulatory activity, anti-inflammatory, antiproliferative and antimicrobial activities have also been attributed to betalains and betalain-rich foods (66, 67). As for many other bioactive compounds, most of these studies are based on *in vitro* cell culture experiments and animal models, whereas human clinical trials as the “Gold-Standard” are still lacking. Furthermore, betanin was also reported to preserve the quality of frozen and refrigerated foods due to its capacity of preventing lipid oxidation (68). However, the use of *Tecticornia* betalains as natural food colorants, their possible synergistic or antagonistic interactions with other food components as well as their potential health benefits need to be investigated in more detail in future studies.

### Vitamin C

Vitamin C was found between 20.5 mg/100 g DW (*T. halocnemoides* 4) and 55.2 mg/100 g DW (*T. halocnemoides* 2), which was in the same range as reported for green tea leaf powder [60 mg/100 g DW] (69). Furthermore, a 200 g serve of fresh *T. halocnemoides* 2 (taking the moisture content of 77% into account) would deliver 56% of the recommended dietary intake (RDI) for vitamin C, which is 45 mg/day for adults in Australia (38).

### Anti-nutrients

The determined anti-nutrients were all lower than those in spinach, except for hydrolysable tannins in *T. halocnemoides* 2, 3, 5, and 6, and phytate in *T. halocnemoides* 3 (Table 6). Furthermore, all *Tecticornia* species had considerably lower levels of saponins compared to Gumby Gumby [*Pittosporum angustifolium*; 1,590-3,645 mg OE/100 g DW; (34)], another plant with functional properties endemic to Australia.

**Table 6:** Anti-nutrients in the studied *Tecticornia* species.

**Plant species**	***T. halocnemoides* 1**	***T. halocnemoides* 2**	***T. halocnemoides* 3**	***T. halocnemoides* 4**	***T. indica* 5**	***T. halocnemoides* 6**	***Spinach***
Hydrolysable Tannin (mg TAE/g DW)	1.2 ± 0.3^a^	7.4 ± 0.5^c^	4.0 ± 0.9^b^	1.4 ± 0.3^a^	3.8 ± 0.4^b^	3.5 ± 0.5^b^	1.5 ± 0.6^a^
Trypsin Inhibitor (TUI/mg DW)	0.3 ± 0.0^a^	1.3 ± 0.0^e^	0.9 ± 0.1^d^	0.3 ± 0.0^a^	0.6 ± 0.0^c^	0.4 ± 0.0^b^	3.5 ± 0.3^f^
Total Saponin Content (mg OE/100 g DW)	496.7 ± 41.3^bc^	571.5 ± 63.0^c^	158.7 ± 38.1^a^	336.0 ± 27.8^ab^	1035.2 ± 102.1^d^	340.6 ± 24.4^ab^	2245.7 ± 148.4^e^
Phytate Content (mg PA/g DW)	15.4 ± 0.1^b^	6.0 ± 0.2^a^	46.2 ± 1.4^c^	6.0 ± 0.2^a^	14.9 ± 0.5^b^	6.0 ± 0.1^a^	46.0 ± 1.1^c^

*Values are means ± SD (n = 3); Means not sharing a common superscript (a–f) within a row are significantly (p < 0.05) different*.

## Conclusions

To the best of our knowledge, this is the first comprehensive evaluation of the nutritional composition of Australian indigenous edible halophytes, grown in different (sub)locations in the Kimberly Region, Western Australia. The results demonstrated the nutritional potential of these *Tecticornia* species in terms of fiber, favorable fatty acid profile (PUFA/SFA ratio), natural pigments and antioxidant capacity. However, future studies are warranted to elucidate the complete nutritional profile, including vitamins (except vitamin C), individual polyphenols and other non-betalain phytochemicals. Furthermore, *in vitro* bioaccessibility and *in vivo* bioavailability studies, together with sensory trials and product development are crucial, to fully understand the nutritional value of these “unique” edible plants for consumers and industry.

## Data Availability Statement

The original contributions generated in the study are included in the article/supplementary material, further inquiries can be directed to the corresponding author/s.

## Author Contributions

All authors contributed extensively to the manuscript and gave final approval for publication.

## Conflict of Interest

The authors declare that the research was conducted in the absence of any commercial or financial relationships that could be construed as a potential conflict of interest.
